# Endoplasmic reticulum stress inhibition attenuates hypertensive chronic kidney disease through reduction in proteinuria

**DOI:** 10.1038/srep41572

**Published:** 2017-02-02

**Authors:** Zahraa Mohammed-Ali, Chao Lu, Mandeep K. Marway, Rachel E. Carlisle, Kjetil Ask, Dusan Lukic, Joan C. Krepinsky, Jeffrey G. Dickhout

**Affiliations:** 1 Department of Medicine, Division of Nephrology, McMaster University and St. Joseph’s Healthcare Hamilton, Hamilton, Ontario, Canada; 2 Department of Medicine, Division of Respirology, McMaster University and St. Joseph’s Healthcare Hamilton, Hamilton, Ontario, Canada; 3 Department of Pathology and Molecular Medicine, McMaster University and St. Joseph’s Healthcare Hamilton, Ontario, Canada

## Abstract

Endoplasmic reticulum (ER) stress is implicated in chronic kidney disease (CKD) development in patients and in animal models. Here we show that ER stress inhibition through 4-phenylbutyric acid (4-PBA) administration decreases blood pressure, albuminuria, and tubular casts in an angiotensin II/deoxycorticosterone acetate/salt murine model of CKD. Lower albuminuria in 4-PBA-treated mice was associated with higher levels of cubilin protein in renal tissue membrane fractions. 4-PBA decreased renal interstitial fibrosis, renal CD3^+^ T-cell and macrophage infiltration, mRNA expression of TGFβ1, Wnt signaling molecules, and ER stress-induced pro-inflammatory genes. CHOP deficient mice that underwent this model of CKD developed hypertension comparable to wild type mice, but had less albuminuria and tubular casts. CHOP deficiency resulted in higher nephrin levels and decreased glomerulosclerosis compared to wild type mice; this effect was accompanied by lower macrophage infiltration and fibrosis. Our findings portray ER stress inhibition as a means to alleviate hypertensive CKD by preserving glomerular barrier integrity and tubular function. These results demonstrate ER stress modulation as a novel target for preserving renal function in hypertensive CKD.

Chronic kidney disease (CKD) can result from a variety of disorders that affect the structure and function of the kidney, and it is a rising global health concern due to its increasing prevalence (8–16%) worldwide^1^. There is presently no cure for CKD and the treatments available are primarily aimed at halting disease progression. Further, CKD can result in end stage renal disease (ESRD) and is associated with complications, such as cardiovascular disease, cognitive decline, anaemia, and mineral and bone disorders^1^. Individuals that progress to ESRD require renal replacement therapy that involves dialysis and/or renal transplants that are extremely costly, $35 billion per year in the United States of America. As well, access to renal replacement therapy is limited in developing and third world countries, causing 1 million people to die annually from ESRD^2^.

The endoplasmic reticulum (ER) is responsible for the synthesis of transmembrane, secretory, and ER lumenal proteins^3^. During diseased states there may be a disruption of ER proteostasis, whereby there is an imbalance between protein folding capacity and protein folding demand^4^. The accumulation of misfolded proteins in the ER is known as ER stress and leads to the activation of the unfolded protein response (UPR). The UPR triggers the dissociation of GRP78 from three known transmembrane sensors in the ER: activating transcription factor 6 (ATF6), inositol-requiring enzyme I (IRE1), and PKR (double-stranded RNA-dependent protein kinase)-like ER protein kinase (PERK). The activation of these signal transduction pathways can result in cell death, inflammation, and fibrosis in the context of CKD^5^,^6^. ER stress has been shown to play a role in the pathogenesis of various renal diseases in humans^3^. The upregulation of key UPR markers has been observed in patients with primary glomerular disease^7^,^8^,^9^ and diabetic nephropathy^10^, and has been strongly associated with proteinuria^11^.

Our central hypothesis in this study is that inhibition of ER stress impedes CKD progression. In our experiments, we utilized two methods to inhibit ER stress, pharmacologically by dietary administration of 4-phenylbutyric acid (PBA) and genetically through the disruption of CHOP, a pro-apoptotic transcription factor that is induced by ER stress.

## Results

### 4-PBA inhibits hypertensive proteinuria in a model of CKD

**Figure 1:**
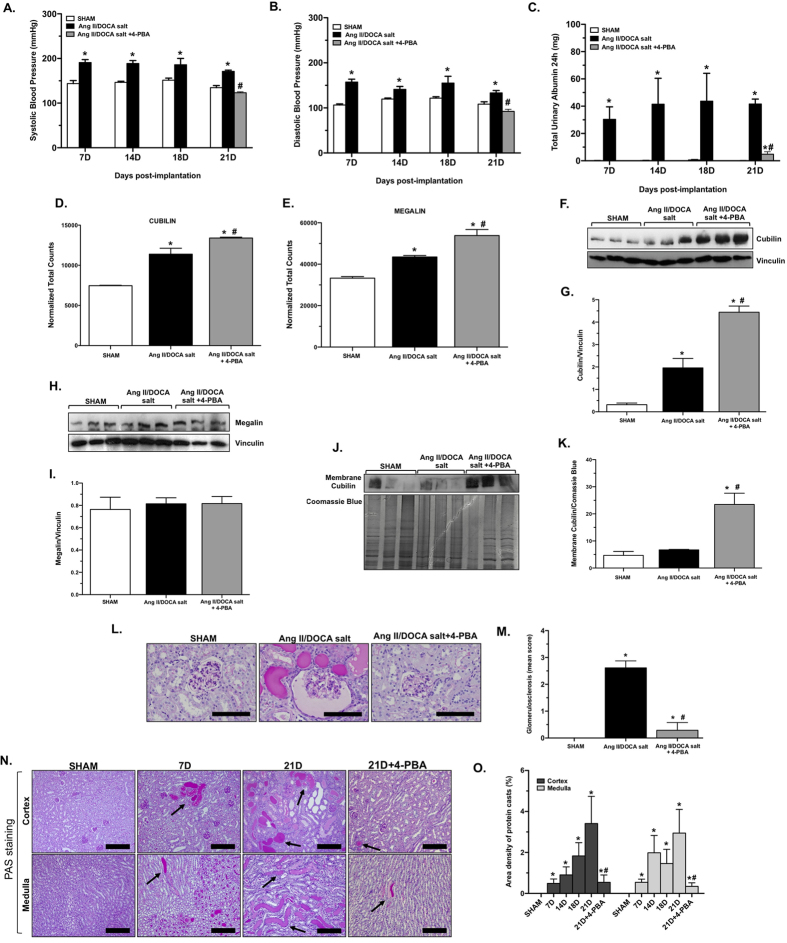
Effect of 4-PBA treatment on the development of hypertensive proteinuria. Mice treated with Ang II/DOCA salt showed a significant increase in (**A**) systolic and (**B**) diastolic blood pressures at the day 7 (7D), 14 (14D), 18 (18D) and 21 (21D) time points (n = 5 per group) compared to their corresponding SHAM controls (n = 3–5 per group). Ang II/DOCA salt animals treated with 4-PBA (n = 5) did not show any change in (A) systolic and (B) diastolic blood pressure. (**C**) Total 24 h urinary albumin excretion was significantly increased by Ang II/DOCA salt at all time points, 7D (n = 5), 14D (n = 5), 18D (n = 4) and 21D (n = 6) compared to SHAM controls (n = 5–6), and was significantly reduced by 4-PBA treatment (n = 6). Normalized total counts for endocytic receptor mRNA (**D**) cubilin and (**E)** megalin show a significant increase with Ang II/DOCA salt (n = 3) treatment, and a further significant increase with 4-PBA treatment (n = 4) compared to SHAM controls (n = 3). (**F,G)** Protein levels of cubilin in whole kidney tissue lysates were significantly increased with Ang II/DOCA salt treatment (n = 3) and showed significantly higher levels with 4-PBA treatment (n = 3). (**H,l**) There was no change in kidney megalin protein levels with Ang II/DOCA salt treatment or 4-PBA treatment (n = 3 per group). (**J,K**) Membrane extracts from kidney tissue lysates showed significantly increased levels of membrane-embedded cubilin in the Ang II/DOCA salt group treated with 4-PBA (n = 3 per group). (**L,M**) 4-PBA significantly decreased glomerulosclerosis in Ang II/DOCA salt mice (n = 3 per group). (**N,O**) An increase in protein cast formation (arrows) was observed in Ang II/DOCA salt mice at all time points (n = 4–5 per group) compared to their respective SHAMs (n = 5–6), and is decreased with 4-PBA treatment (n = 6). 21D SHAM (n = 5) is used as a representative image for all shams. *Denotes significantly different than the corresponding SHAM; ^#^indicates significantly different in the D21 Ang II/DOCA salt + 4-PBA compared to day 21 Ang II/DOCA salt. Scale bars = 100 μm in (**L**) and 200 μm in (**N**).

This CKD model produced a significant increase in systolic (Fig. 1A) and diastolic blood pressure (Fig. 1B) at day 7 post-angiotensin (Ang) II/deoxycorticosterone acetate (DOCA) salt treatment, continuing until sacrifice at day 21. Hypertension was completely inhibited by 4-PBA treatment, as measured on day 21. An increase in albuminuria was observed at all time points in response to Ang II/DOCA salt treatment. 4-PBA treatment significantly reduced 24 h urinary albumin excretion at day 21 (Fig. 1C). To investigate why 4-PBA treatment reduced albuminuria, we examined megalin and cubilin, two multi-ligand endocytic receptors, responsible for tubular protein reabsorption^12^. Proteinuric kidney disease places an increased demand for synthesis of these receptors in the ER^13^. Since 4-PBA alleviates ER stress by aiding in protein folding, we hypothesized that mice treated with 4-PBA could produce higher amounts of megalin and cubilin in response to the Ang II/DOCA salt-induced CKD. Therefore, we measured megalin and cubilin expression at the RNA and protein level. Nanostring nCounter assay analysis showed an increase in megalin (Fig. 1D) and cubilin (Fig. 1E) mRNA in response to Ang II/DOCA salt treatment. 4-PBA treatment resulted in an even higher level of megalin and cubilin mRNA, suggesting that the action of 4-PBA increased endocytic receptor transcription. Western blotting showed an increase in cubilin protein expression (Fig. 1F,G) in response to Ang II/DOCA salt treatment, with higher levels in 4-PBA-treated mice; however, no change was found in megalin protein levels (Fig. 1H,I). To decipher whether this increase in cubilin protein reached the plasma membrane to become a functional endocytic receptor, we extracted plasma membrane fractions from our experimental groups and measured membrane cubilin expression. Figure 1J,K showed significantly increased cubilin in plasma membrane lysates from 4-PBA-treated animals compared to untreated animals in response to Ang II/DOCA salt. In attempting to measure megalin in these same membrane fractions, we were not able to obtain a reliable band in the 600 kDa range, the known molecular weight for the protein. The effect of albuminuria on renal pathology was examined through periodic acid-Schiff (PAS) staining by scoring glomerular damage and quantifying intratubular protein casts. In AngII/DOCA salt mice, 4-PBA treatment significantly decreased glomerulosclerosis (Fig. 1L,M) and significantly reduced intratubuluar protein cast formation in both the cortex and the medulla (Fig. 1N,O).

### 4-PBA inhibits the UPR in a model of CKD

**Figure 2:**
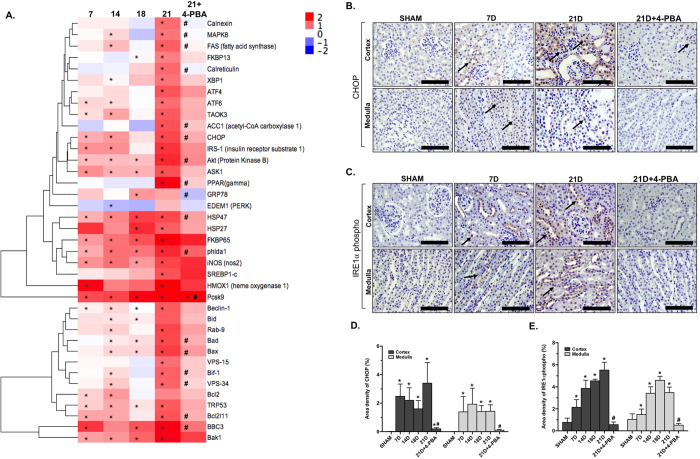
Effect of 4-PBA treatment on the UPR activation. (**A**) Hierarchical clustering of ER stress and apoptotic genes is demonstrated using a heat map. Fold changes represent the changes in gene expression with Ang II/DOCA salt treatment at days 7 (7D; n = 4), 14 (14D; n = 5), 18 (18D; n = 3), 21 (21D; n = 5) and 21D + 4-PBA (n = 5) compared to their respective sham-operated controls. (**B,D**) CHOP and (**C,E**) phosphorylated IRE1α staining (60X) in the kidney was increased by Ang II/DOCA salt (n = 5 for 7D, 14D and 21D, n = 4 for 18D), as indicated by arrows, and inhibited by 4-PBA treatment (n = 5). The 21D SHAM group (n = 5 for cortex, n = 3 for medulla) is representative of all shams. *indicates that the Ang II/DOCA salt group is significantly different than corresponding SHAM at that time point; ^#^indicates that the 21D + 4-PBA is significantly different than the 21D Ang II/DOCA salt group. Scale bars = 100 μm in (**B**) and (**C**).

The NanoString nCounter determined the fold change of UPR gene expression over 21 days of Ang II/DOCA/salt-induced CKD (Fig. 2A). Fold changes for the 4-PBA-treated Ang II/DOCA salt mice were compared to sham controls that were also sacrificed at day 21. Collagen-folding chaperones, such as *Hsp47* and *Fkbp65*^14^,^15^, were induced in the Ang II/DOCA salt-treated mice at day 7 and remained elevated until the endpoint of the study. *Hmox-1*, a gene known to be upregulated during ER stress^16^, and an important marker of human CKD progression^17^ was shown to be significantly induced by the AngII/DOCA salt model at an early stage. Our CKD mouse model also showed an increase in apoptosis and authophagy genes that are induced by IRE1α phosphorylation and mediated by *Ask1* during ER stress. These genes include *Bcl2, Bad, Bax, Bak1, Bbc3, Trp53,* and *Beclin-1*^18^,^19^. As well, apoptotic genes that have been shown to be directly induced by ER stress, *Chop* and *phlda1,* are increased in our CKD model^20^. 4-PBA treatment inhibited the activation of UPR genes in response to Ang II/DOCA salt at the 21-day time point. *Pcsk9* was the only gene that was significantly induced by 4-PBA and was significantly higher in the 4-PBA-treated group compared to Ang II/DOCA salt mice without 4-PBA treatment. To examine the UPR activation in our CKD model, kidneys were stained for CHOP (Fig. 2B) and phosphorylated IRE1α (Fig. 2C). Quantification of the staining showed an increase in CHOP (Fig. 2D) and phosphorylated IRE1α (Fig. 2E) at all time points; both CHOP and phosphorylated IRE1α were inhibited by 4-PBA treatment (Fig. 2D,E).

### 4-PBA attenuated renal interstitial fibrosis in Ang II/DOCA salt-induced CKD

**Figure 3:**
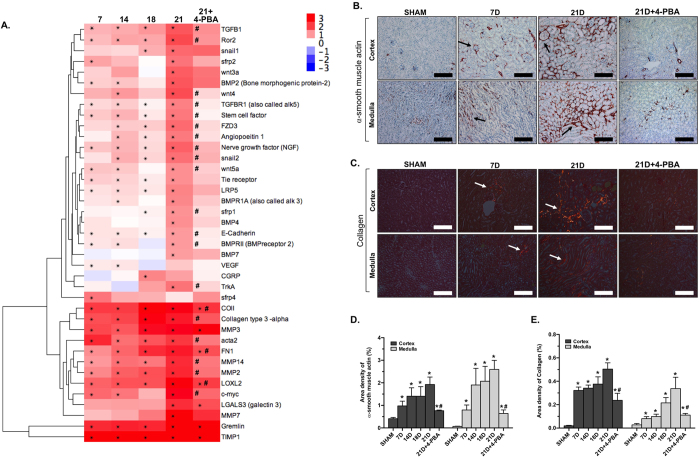
Effect of 4-PBA treatment on renal fibrosis. (**A**) Hierarchical clustering of fibrotic genes is demonstrated in a heat map. Fold changes represent the changes in gene expression with Ang II/DOCA salt treatment at days 7 (7D; n = 4), 14 (14D; n = 5), 18 (18D; n = 3), 21 (21D; n = 5) and 21D + 4-PBA (n = 5) compared to their respective sham-operated controls. (**B,D**) α-smooth muscle actin staining (arrows) (20X) is increased at all timepoints (n = 4 per group). (**C,E**) Collagen deposition (arrows), demonstrated using picrosirius red staining (20X), was significantly elevated at all timepoints (n = 5 for 7D, 14D and 18D and n = 4 for 21D). 4-PBA treatment (n = 3) significantly decreased both collagen and α-smooth muscle actin deposition in response to Ang II/DOCA salt. Only the representative sham, 21D SHAM group (n = 4 for cortex, n = 3 for medulla), is shown. *indicates that the Ang II/DOCA salt treatment is significantly different than corresponding sham at that time point; ^#^indicates that the 21D + 4-PBA group is significantly different than the 21D Ang II/DOCA salt group. Scale bars = 200 μm in (**B**) and (**C**).

A Nanostring nCounter assay was performed to evaluate the fold changes in the expression of fibrosis genes illustrated in a heat map (Fig. 3A). Genes encoding extracellular matrix (ECM) proteins collagen (*Col1a1, Col3a1*) and fibronectin (*Fn1*), myofibroblast marker α-smooth muscle actin (*Acta2*), as well as regulators of the ECM deposition process such as E-cadherin, *Mmp2, Mmp3, Mmp14, Timp1,* are shown to be upregulated early in CKD development at day 7 and continue to exhibit high levels of expression through all time points. The ECM deposition process is initiated by TGF-β and Wnt signaling pathways in various animal models of kidney disease, as well as in human CKD^21^,^22^. Our Ang II/DOCA salt model resulted in an early upregulation of TGF-β signaling and non-canonical Wnt signaling (*Wnt5a-Ror2*) as early as day 7 in the model and these pathways were significantly lowered with 4-PBA treatment. Although 4-PBA-treated mice still experienced a significant increase in pro-fibrotic genes *Col1a1, Mmp3, Fn1, Loxl2, Lgals3, Gremlin,* and *Timp-1*, there was an overall decrease in ECM components and fibrotic mediators. Kidneys were stained with α-smooth muscle actin (Fig. 3B) and picrosirius red (Fig. 3C) to examine renal fibrosis. Analysis of α-smooth muscle actin expression (Fig. 3D) and collagen deposition (Fig. 3E) demonstrated that ECM deposition was increased at day 7 and continued rising until sacrifice at day 21. Although some α-smooth muscle actin and collagen deposition was observed in 4-PBA-treated Ang II/DOCA salt mice, it was significantly lower than Ang II/DOCA salt mice (Fig. 3D,E).

### 4-PBA treatment attenuated inflammation in Ang II/DOCA salt-mediated CKD

**Figure 4:**
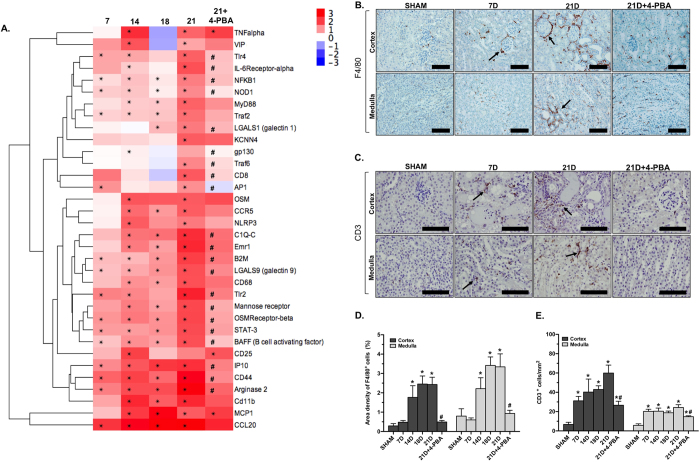
Effect of 4-PBA treatment on renal inflammation. (**A**) A heat map demonstrates hierarchical clustering of inflammatory genes. Fold changes represent the changes in gene expression with Ang II/DOCA salt treatment at days 7 (7D; n = 4), 14 (14D; n = 5), 18 (18D; n = 3), 21 (21D; n = 5) and 21D + 4-PBA (n = 5), compared to their respective sham-operated controls. **(B,D**) F4/80 staining (40X; arrows) shows an increase in F4/80^+^ macrophage infiltration at 14D (n = 4), 18D (n = 5) and 21D (n = 5), but not at 7D (n = 6). (**C,E**) CD3 staining (60X; arrows) demonstrates significant T-cell infiltration at all time points (n = 4 per group). 4-PBA treatment (n = 3) significantly decreased macrophage and T-cell infiltration in response to Ang II/DOCA salt. Only the representative 21D SHAM is included in the figure (n = 5 for F4/80 staining, n = 4 for CD3 staining). *indicates that the Ang II/DOCA salt is significantly different than corresponding sham at that time point (n = 4–5 per group); ^#^indicates that the 21D + 4-PBA is significantly different than the 21D Ang II/DOCA salt group. Scale bars = 100 μm in (**B**) and (**C**).

Fold changes in the expression of inflammatory genes are demonstrated in a heat map (Fig. 4A). Only a few genes appeared to be regulated at day 7 in the inflammatory gene codeset, whereas almost all inflammatory genes were upregulated by day 21. This temporality in response allowed us to isolate potential initiators of CKD development. These include pattern recognition receptors (PRRs) such as *Tlr2, Tlr4,* and *Nod-1* that have been shown to be associated with various models of kidney injury^23^,^24^. Transcription factors *NF-κB, AP-1*, and *Stat3,* which have been reported to be induced by ER stress^25^,^26^ were also significantly increased at day 7 in our model. The NanoString data showed an increase in chemokines *IP-10* and *Ccl20*, which are known to attract various immune cells to the site of tissue injury. Therefore, staining for F4/80 and CD3, well-established cell-surface markers of macrophages (Fig. 4B) and T-cells (Fig. 4C), respectively, were used to assess inflammation in kidney micrographs. This CKD model resulted in significantly increased macrophage infiltration at day 14, 18 and 21 (Fig. 4D) and T-cell infiltration at all time points (Fig. 4E). The quantification of F4/80 staining shows that 4-PBA treatment completely inhibited macrophage infiltration in response to Ang II/DOCA salt treatment (Fig. 4D). However, 4-PBA-treated mice still experienced a certain level of T-cell infiltration in response to Ang II/DOCA/salt, although it was significantly lower than mice without 4-PBA treatment (Fig. 4E).

### CHOP deficiency inhibits proteinuria but not hypertension in a CKD model

**Figure 5:**
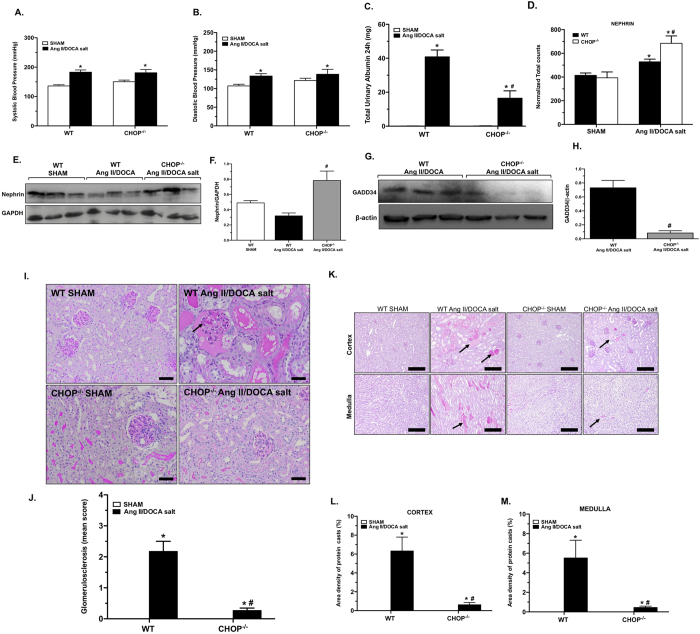
Effect of CHOP knockout on the development of hypertensive proteinuria. Both wild type (WT) (n = 6) and CHOP^−/−^ (n = 5) mice treated with the CKD model experienced an increase in (**A**) systolic and (**B**) diastolic blood pressures, compared to their respective SHAM controls (n = 6 for WT SHAM and n = 5 for CHOP^−/−^ SHAM). (**C**) Although CHOP^−/−^ mice (n = 5) showed an increase in albuminuria with CKD development compared to CHOP^−/−^ SHAM mice (n = 6), they had significantly lower albuminuria compared to WT mice (n = 7). (**D**) Nephrin mRNA levels increase with Ang II/DOCA salt treatment compared to sham controls (n = 3 per group) and are significantly higher in CHOP^−/−^ animals treated with Ang II/DOCA salt (n = 5) compared to WT Ang II/DOCA salt animals (n = 5). (**E,F**) Protein levels of nephrin were significantly increased in whole kidney tissue lysates (n = 3 per group) in CHOP^−/−^ mice. (**G,H**) Significantly lower levels of GADD34 were observed in CHOP^−/−^ mice. (**I,J**) CHOP^−/−^ mice (n = 5) showed significantly lower glomerular sclerosis (arrows) compared to WT mice (n = 5) in response to Ang II/DOCA salt. (**K–M**) Protein cast formation (arrows; 20X) demonstrates less damage in CHOP^−/−^ mice (n = 5) in response to Ang II/DOCA salt compared to treated WT mice (n = 5). There was no difference in glomerular score or protein cast formation between CHOP^−/−^ sham (n = 6) and WT sham (n = 6). *indicates significantly different than the respective sham-operated controls; ^#^indicates significant difference between the CHOP^−/−^ Ang II/DOCA salt mice compared to WT Ang II/DOCA salt mice. Scale bars = 50 μm in (**I**) and 200 μm (**K**).

Since CHOP is a key transcription factor in the UPR and was upregulated at the earliest time point, day 7, in our model, we examined the effect of genetic disruption of CHOP on CKD severity. In response to the Ang II/DOCA/salt model, CHOP^−/−^ mice experienced an increase in both systolic (Fig. 5A) and diastolic blood pressure (Fig. 5B), which was comparable to wild type (WT) mice. However, Ang II/DOCA salt-treated CHOP^−/−^ mice showed a significantly lower level of albuminuria compared with WT mice (Fig. 5C). Therefore, we explored possible mechanisms for the decreased albumin levels found in CHOP^−/−^ mice. Reports have established that eIF2α phosphorylation during ER stress results in general translation attenuation and CHOP induction. CHOP leads to the activation of GADD34, which is able to recover protein translation by dephosphorylating eIF2α^27^. During translation attenuation proteins with short open reading frames such as ATF4 are preferentially translated^28^. Nephrin, an integral component of the podocyte slit diaphragm, is another protein that is preferentially translated during eIF2α phosphorylation^28^. Our experiments show an increase in nephrin in response to our CKD model and a significantly higher amount of nephrin in CHOP^−/−^ mice treated with Ang II/DOCA salt than in WT mice at both the mRNA (Fig. 5D) and the protein level (Fig. 5E,F). As well, our data shows a significantly lower level of GADD34 in CHOP^−/−^ mice (Fig. 5G,H), confirming our suggested mechanism. Since nephrin is important in maintaining the renal filtration barrier, we then investigated the effect of CHOP deficiency on the maintenance of glomerular structure and renal pathology. Glomerular scoring showed a significantly lower degree of glomerular damage in CHOP^−/−^ mice that underwent the Ang II/DOCA salt model compared to WT mice (Fig. 5I,J). PAS staining demonstrated a significantly lower level of protein cast formation in the cortex and medulla of CHOP^−/−^ mice compared to WT mice (Fig. 5K,L,M) in response to Ang II/DOCA salt.

### CHOP deficiency inhibits the UPR, inflammation, and fibrosis in CKD

**Figure 6:**
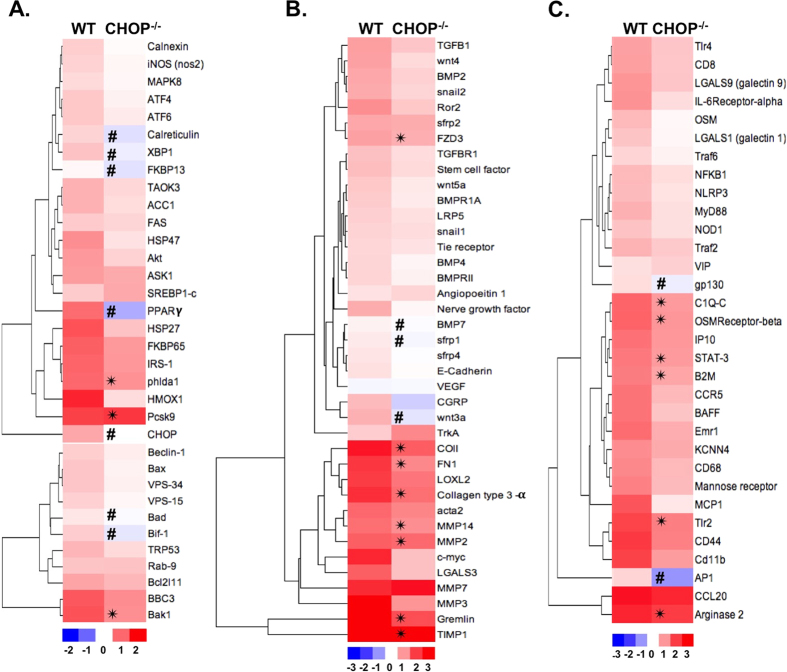
Effect of CHOP deficiency on UPR, fibrosis, and inflammatory gene expression. Heat maps demonstrate hierarchical clustering of (**A**) UPR, (**B**) fibrotic, and (**C**) inflammatory genes. “WT” column represents fold changes in gene expression with Ang II/DOCA salt treatment in wild type (WT; n = 6) compared to WT SHAM (n = 3). “CHOP^−/−^” column represents fold changes in treated CHOP^−/−^ mice (n = 5) compared to CHOP^−/−^ sham mice (n = 3). Genes included in these heat maps are those that were significantly regulated in WT Ang II/DOCA salt-treated mice compared to WT sham at day 21. CHOP deficiency attenuated the upregulation of important pathways underlying CKD. *indicates that Ang II/DOCA salt CHOP^−/−^ is significantly different than corresponding sham in CHOP^−/−^ mice; ^#^indicates that the gene is regulated in the opposite direction in CHOP^−/−^ Ang II/DOCA salt mice compared to WT Ang II/DOCA salt mice.

**Figure 7:**
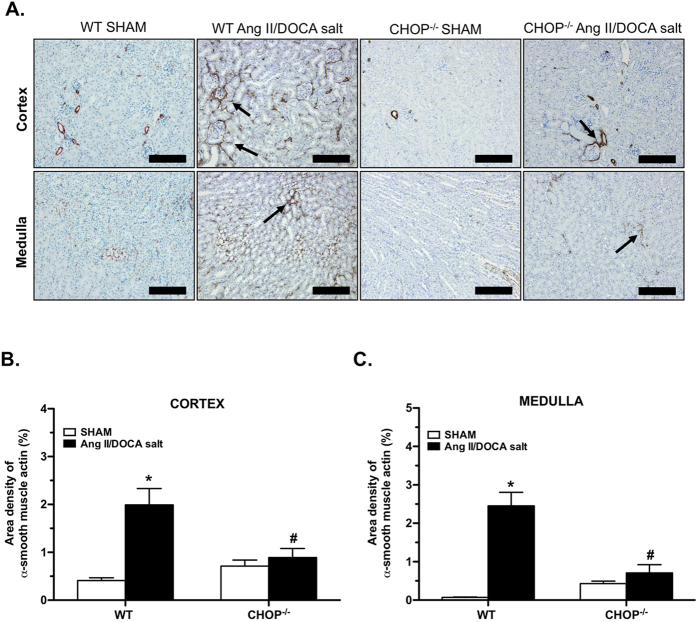
Effect of CHOP deficiency on the development of fibrosis. (**A–C**) α-smooth muscle actin staining (arrows; 20X) is reduced with CHOP deficiency. Extracellular matrix deposition was significantly increased by Ang II/DOCA salt in wild type (WT) mice (n = 5), but not in CHOP^−/−^ mice (n = 5). *indicates that the Ang II/DOCA salt is significantly different than corresponding sham; ^#^indicates that the CHOP^−/−^ mice are significantly different than WT mice. Scale bars = 200 μm in (**A**).

**Figure 8:**
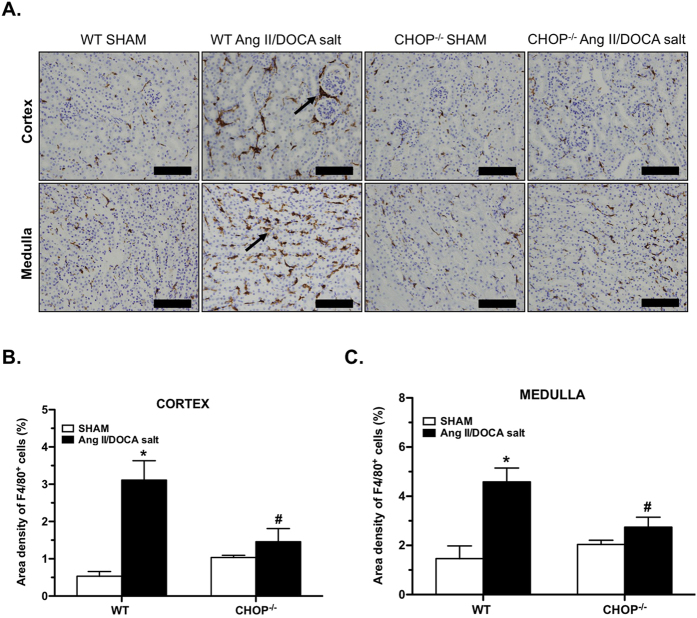
Effect of CHOP deficiency on the development of inflammation. (**A–C**) F4/80 staining (arrows; 40X) demonstrates that macrophage infiltration was significantly increased by Ang II/DOCA salt in wild type (WT; n = 4) but not in CHOP^−/−^ mice (n = 5), compared to their corresponding sham controls (n = 4). *indicates that the Ang II/DOCA salt mice are significantly different than corresponding sham; ^#^indicates that the CHOP^−/−^ mice are significantly different than WT mice. Scale bars = 100 μm in (**A**).

CHOP deficiency reduced the number of genes regulated in response to Ang II/DOCA salt. Lower levels of UPR (Fig. 6A), fibrotic (Fig. 6B), and inflammatory (Fig. 6C) genes were induced by the Ang II/DOCA salt model in the CHOP^−/−^ mice compared to WT mice. Of note, *Pcsk9* is higher in the kidney in CHOP^−/−^ mice treated with AngII/DOCA salt. Some UPR and apoptosis/autophagy genes were regulated in the opposite direction in CHOP^−/−^ mice treated with Ang II/DOCA salt compared to WT, such as *PPARγ, Bad, Bif-1, Calreticulin,* and *Fkbp13*. Similarly, transcription factor *AP-1* and transmembrane protein *gp130* also experienced downregulation in CHOP^−/−^ mice treated with Ang II/DOCA salt, as opposed to upregulation in WT mice. Although certain ECM deposition components such as *Col1A1, Col3A1, Fn1*, showed a significant increase in CHOP^−/−^ mice treated with Ang II/DOCA salt, the level of increase was attenuated. α-smooth muscle actin staining of renal tissue was significantly lower in the cortex and medulla of CHOP^−/−^ mice treated with Ang II/DOCA salt than in WT mice (Fig. 7A,B). Similarly, area density of F4/80 staining showed significantly lower macrophage infiltration in CHOP^−/−^ mice treated with Ang II/DOCA salt compared with WT mice (Fig. 8A,C).

### The UPR is activated in human hypertensive nephrosclerosis

**Figure 9:**
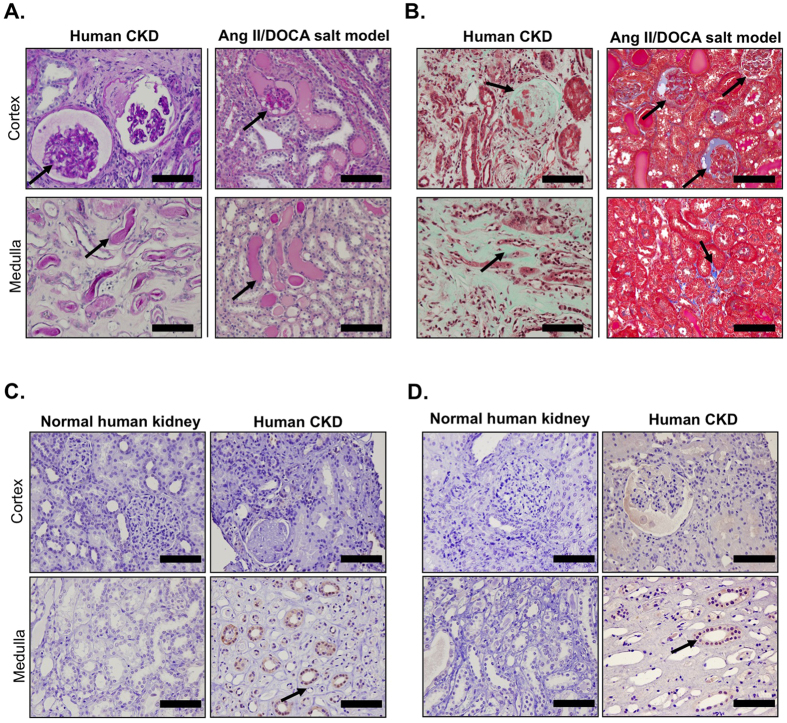
Similarities in pathology between human and murine chronic kidney disease. This Ang II/DOCA salt mouse model of chronic kidney disease (CKD) produces (**A**) protein cast formation, demonstrated by periodic acid-Schiff staining, and (**B**) fibrosis, demonstrated by Masson’s trichrome staining, at a comparable level to the kidney damage found in hypertensive nephrosclerotic human patients. Human CKD patients (n = 2) show an increase in medullary (**C**) phosphorylated IRE1α and (**D**) CHOP staining (arrows) compared to non-CKD patients. Scale bars = 100 μm in (**A–D**).

In order to translate our work into human CKD, we studied the renal pathology in patients with hypertensive nephrosclerosis in comparison to renal pathology in our mouse model. The similarities in pathology are demonstrated in terms of glomerular damage between our mouse model and CKD patients. Further, protein cast formation was present in both the cortex and the medulla of CKD patients, similar to the Ang II/DOCA salt mouse model (Fig. 9A). Masson’s trichrome staining showed glomerular and renal interstitial fibrosis in both human CKD and the Ang II/DOCA salt model (Fig. 9B). Likewise, an increase in key UPR genes, specifically phosphorylated IRE1α (Fig. 9C) and CHOP (Fig. 9D), was found in the medullae of biopsies of human hypertensive CKD.

## Discussion

In this study, we investigated the temporal relationships between the inflammatory, fibrotic, and UPR pathways in CKD. Our results implicate the UPR as an initiator of CKD, since key ER stress genes, *Grp78, Chop, Atf6* and *phlda1* were upregulated early in the disease process. ER stress-induced apoptotic and autophagy genes, *Ask1, Bad, Bax, Bcl2, Bbc3* and *Beclin-1*^18^,^19^, were also upregulated early in our model. This model shows increased albuminuria and protein cast formation early in CKD development. These results indicate albuminuria and renal pathology occurs at the same time as UPR activation and are in accordance with previous reports^11^,^29^.

4-PBA is a small molecular chaperone that alleviates ER stress by aiding in the protein folding in the ER^30^,^31^. We found 4-PBA significantly reduced albuminuria and CKD progression in our model while inhibiting the upregulation of key UPR genes. This result is in agreement with previous studies, where 4-PBA administration has been reported to decrease urinary protein excretion and glomerular damage in streptozotocin-induced diabetic nephropathy^30^,^32^. As well, 4-PBA has been shown to decrease outer medullary stripe damage in tunicamycin-induced acute kidney injury (AKI)^33^. Our experiments are the first to draw a link between the effect of 4-PBA treatment and albumin reabsorption mediated by megalin and cubilin in renal proximal tubules. In addition to abnormalities in the glomerular filtration barrier, defective tubular reabsorption may also contribute to albuminuria^34^. In our study, 4-PBA treatment resulted in lower albuminuria, as well as a significant increase in megalin and cubilin mRNA. Protein analysis showed an increase in cubilin in both whole kidney tissue lysates, as well as plasma membrane extracts in 4-PBA-treated mice. In line with our findings, a recent study^35^ showed a six-fold increase in urinary albumin excretion in cubilin-deficient mice. Similarly, patients with defective cubilin, resulting in Imerslund-Gräsbeck disease, also have higher urinary excretion of albumin, along with other ligands of cubilin^36^,^37^. It appears that 4-PBA increased the folding and delivery of cubilin to the apical membrane of the proximal tubule allowing for a greater reabsorption of albumin, thereby reducing 24 h albumin excretion. However, further experiments are necessary to conclude that 4-PBA increased cubilin directly through its chaperone activity, since 4-PBA treatment augmented both mRNA and protein cubilin levels. Therefore, the significantly lower albuminuria in 4-PBA-treated mice could be attributed to the increased cubilin levels in these mice.

Albuminuria is an important risk factor for CKD progression^38^,^39^. Albumin excretion associated with renal injury has been shown to cause TGF-β activation^40^,^41^,^42^. Our study shows increased TGF-β1 signalling, which stimulates resident fibroblasts, myofibroblasts, and tubular epithelial cells to produce ECM components, collagens type I and III, FN1, and α-smooth muscle actin, resulting in fibrosis^21^. 4-PBA treatment inhibited α-smooth muscle actin and collagen protein deposition in response to Ang II/DOCA/salt. Our experiments, along with others on the unilateral ureteral obstruction (UUO) model, have shown 4-PBA treatment to impart protection against apoptosis and renal interstitial fibrosis^43^,^44^, suggesting a causative role for ER stress in renal fibrosis. Further, in a study by El Karoui *et al*.^45^. 4-PBA treatment resulted in decreased tubular lesions, interstitial fibrosis and tubular apoptosis in WT1^+/*mut*^ mice which develop glomerular disease due to a mutation in WT1, an important regulator of podocyte function. In this study, 4-PBA was also administered in a model of doxorubicin-induced nephropathy where it caused a reduction in protein cast formation, apoptosis and ER stress in the kidney^45^. The effect of 4-PBA was partly linked to its inhibition of Lipocalin 2, a gene requiring calcium release-induced ER stress for its induction. Lipocalin 2 induction was shown to generate tubular apoptosis and renal lesions. Interestingly, the authors demonstrated that albumin is able to activate calcium-dependent ER stress. They speculate that this may occur through albumin binding to megalin in a calcium-dependent manner thereby triggering intracellular ER calcium release and ER stress^45^. Our study adds to this data by showing further association between ER stress inhibition and decreased albuminuria through the increase in membrane cubilin available for albumin reabsorption.

The IRE1α and PERK pathways of the UPR result in inflammation through the activation of *NF-κB* and *AP-1*, transcription factors responsible for the upregulation of cytokines, chemokines, complement, and acute phase proteins^6^,^25^. In our study, both *NF-κB* and *AP-1* were significantly upregulated in our CKD model. Immune cell recruitment was increased with Ang II/DOCA/salt CKD, with an increased infiltration of CD3^ + ^and F4/80^ + ^cells in the kidneys of mice with CKD. Macrophage and T-cell infiltration has been shown in human and animal models of kidney disease and is associated with disease progression and proteinuria^46^. Studies on the streptozotocin model of diabetic nephropathy have shown that in addition to lowering urinary protein excretion, 4-PBA attenuates inflammatory mediators NF-kB and MCP-1^30^,^47^. MCP-1 is responsible for the recruitment of macrophages, and its urinary levels in humans have shown strong association with proteinuria^48^. In fact, MCP-1 deficiency has been shown to prevent renal fibrosis, proteinuria, and macrophage infiltration in various animal models of disease^48^. 4-PBA significantly reduced inflammatory cell infiltration and inflammatory gene expression including NF-kB and MCP-1 in our study. In addition to reducing albuminuria and renal injury, 4-PBA is able to prevent disease progression through its inhibitory effect on pro-inflammatory and pro-fibrotic mediators.

Since CHOP was upregulated early in our CKD model, we examined the efficacy of CHOP knockout to inhibit CKD progression. CHOP^−/−^ mice have previously been studied in the context of renal fibrosis, where they have been shown to protect against apoptosis, fibrosis, and inflammation in UUO and AKI^33^,^44^,^49^,^50^. Our experiments have shown that in response to Ang II/DOCA/salt, CHOP^−/−^ mice exhibit significantly lower albuminuria, glomerular injury, and protein cast formation compared to WT mice. Interestingly, unlike the 4-PBA treatment group, they experienced an increase in blood pressure similar to WT mice.

Nephrin is a key component of the slit diaphragm in glomerular podocytes, which helps maintain the glomerular filtration barrier^51^. Cybulsky *et al*.^28^ provided evidence that, analogous to ATF4, nephrin could be preferentially translated in stressed cells during eIF2α phosphorylation, owing to its short upstream open reading frames. General translation attenuation due to eIF2α phosphorylation is relieved by GADD34, a phosphatase induced by CHOP. Prolonged translation attenuation and subsequently lower levels of GADD34, achieved through CHOP deficiency, would allow increased preferential translation of nephrin during ER stress. Our experiments have shown a significant increase in nephrin mRNA with Ang II/DOCA salt treatment and even higher expression in CHOP^−/−^ mice. At the protein level, however, WT Ang II/DOCA salt mice have similar levels of nephrin as WT sham animals, whereas CHOP^−/−^ mice expressed significantly higher levels of nephrin. Taken together, our findings indicate a link between diminished CHOP expression and improved glomerular permselectivity.

Our experiments show lower ER stress and apoptotic gene expression with CHOP deficiency. UPR genes *Atf6, Atf4, Calnexin* and *Calreticulin,* as well as apoptotic and autophagy genes upregulated by the UPR, *Bax, Bad* and *Beclin-1,* show reduced expression in CHOP^−/−^ mice. The inhibition of UPR genes by CHOP deficiency has been demonstrated previously^49^. CHOP has also been shown to downregulate pro-survival gene *Bcl2*, thereby resulting in the activation of apoptosis via Bax/Bak proteins and Beclin-1^18^,^52^. In addition, CHOP^−/−^ mice had an overall trend of attenuated expression of fibrotic and inflammatory genes, as compared to their WT counterparts. Upregulation of key inflammatory transcription factors *NF-κB* and *AP-1* was diminished in CHOP^−/−^ mice. In fact, *AP-1* is downregulated with CHOP deficiency; this could be due to the reported ability of CHOP to enhance the transcriptional activation of *AP-1*^53^. The weakened inflammatory gene expression in CHOP^−/−^ mice on Ang II/DOCA salt was also reflected by lower macrophage infiltration. A recent study by Zhang *et al*.^49^. has shown CHOP deficiency reduces fibrosis, macrophage infiltration, and TLR4-NFκB signaling in a unilateral ureteral obstruction model of kidney disease. This group attributed the reduction in fibrosis to the decreased TLR4-NFκB signalling, which results in significantly lower levels of TGF-β activity^49^. Our experiments also show a decrease in renal fibrosis in line with previous reports on CHOP knockouts; CHOP deficiency attenuated *Tlr4, NF-κB,* and *TGF-β* activity in response to Ang II/DOCA salt.

The finding that CHOP and phosphorylated IRE1α are upregulated in patients with hypertensive nephrosclerosis aids in translating our work to human CKD. Both methods of ER stress inhibition, 4-PBA treatment and CHOP knockdown, were shown to decrease renal fibrosis, inflammation, and albuminuria, important predictors of disease progression in humans. Taken together, these studies provide strong support for a potential therapeutic benefit of 4-PBA in patients with hypertensive kidney disease.

## Methods and Materials

### Mouse model of CKD

A combination of uninephrectomy, Ang II/DOCA infusion, as well as salt in the drinking water was used to induce CKD, as previously documented^8^,^54^. Briefly, 10-week-old male mice were subjected to a uninephrectomy under isofluorane anesthesia. Upon recovery, Ang II (1.5 ng/min/g body weight) was administered via osmotic pump along with a 50 mg 21-day release DOCA pellet. Mice were then provided with 1% salt in drinking water and sacrificed at 7, 14, 18 and 21 days. For each time point, a sham operation was performed on a group of mice for both the uninephrectomy and osmotic pump and pellet implantation; these mice were used as controls. 4-PBA (Scandinavian Formulas) was administered in the drinking water upon pump and pellet implantation at 1 g/kg/day to determine if this treatment would affect the endpoint of CKD development for the 21-day group. Dose was adjusted through measurement of water intake in metabolic cages. Mice placed on 4-PBA were also provided with 1% salt in the drinking water while factoring in the contribution of 4-PBA to the sodium content. Age-matched CHOP^−/−^ mice were obtained from a breeding colony held at McMaster University Central Animal Facility along with their wild type littermate controls. The founders of the colony were purchased from Jackson Laboratories (Stock number: 05530). All animal work was done in accordance with and approved by the McMaster University Animal Research Ethics Board.

### Human Kidney Biopsies

Kidney biopsies were obtained from the Department of Pathology at St. Joseph’s Healthcare Hamilton, Canada. Human CKD kidney biopsies were obtained from patients diagnosed with purely hypertensive nephrosclerosis. Uninvolved, normal renal tissue was obtained from kidneys resected from renal cell carcinoma patients. Work on these tissues was approved by the Hamilton Integrated Research Ethics Board.

### Blood pressure measurements and urinalysis

Blood pressure measurements were obtained through the tail cuff method using a CODA blood pressure analyzer (Kent Scientific), as done previously^54^. Metabolic cages were used to collect 24 h urine before the surgical procedure and after 3 weeks on treatment with AngII/DOCA/salt. Albuminuria, an important risk factor for CKD progression^55^,^56^, was assessed using an ELISA (Bethyl Laboratories) to measure mouse urine albumin concentration in 24 h urine.

### Western blotting

Total cell lysates were obtained using 4X SDS lysis buffer with protease inhibitor cocktail (Roche) and phosphatase inhibitor cocktail (Roche) added. Plasma membrane lysates were obtained using the Mem-PER plus kit (ThermoFisher Scientific). Protein levels were determined using BioRad DC Protein Assay (BioRad) for control of protein loading. Tissue lysates were subjected to electrophoretic separation in an SDS-PAGE reducing gel (BioRad). Primary antibodies were detected using appropriate horseradish peroxidase-conjugated secondary antibodies and ECL Western Blotting Detection Reagents (GE Healthcare), as described previously^33^. Nephrin antibody (AF3159, R& D Systems) was diluted 1:10,000, GADD34 antibody (sc-8327, Santa Cruz) was diluted 1:200, cubilin antibody (sc-20609, Santa Cruz) was diluted 1:200, megalin antibody (sc-25470, Santa Cruz) was diluted 1:100, vinculin antibody (Sigma) was diluted 1:500 and β-actin antibody (Sigma) was diluted 1:5000. Results were densitometrically quantified using ImageLab software (BioRad) and expressed as a ratio of loading controls.

### Immunohistochemistry

At sacrifice, renal tissue was fixed in 4% paraformaldehyde and subsequently sectioned using a microtome. To evaluate renal pathology, PAS staining was performed to assess protein cast formation, and picrosirius red (PSR) and α-smooth muscle actin staining was used to assess fibrosis. For PSR staining, kidney tissue sections were processed with saturated picric acid solution and then stained with Sirius red F3B (Colour Index 35782). For T-cell staining, rabbit anti-human CD3 antibody (Dakocytomation) was used at a concentration of 0.6 g/L and a dilution of 1:100 in normal goat serum (Vector). For macrophage staining, rat antibody to F4/80 (ab6640, Abcam) was used at a dilution of 1:30 in normal rabbit serum. For CHOP staining, rabbit anti-GADD153 (sc-575, Santa Cruz) was used at 1:40 dilution. To detect IRE1α phosphorylation, anti-IRE1 (phospho S724) antibody (ab48187, Abcam) was used at a dilution of 1:100. Horseradish peroxidase streptavidin (Vector SA-5004) was used in combination with Nova Red (Vector) and hematoxylin (Sigma) to visualize the stained cells.

### Assessment of renal pathology

Immunohistochemistry sections were imaged using an Olympus BX41 microscope. Images of PSR-stained kidneys were taken using the U-POT polarizer and U-ANT analyzer (Olympus) for transmitted light. The diaphragm and polarizer allowed the detection of birefringence of collagen fibres. All other stained kidneys were imaged without the polarizer and analyzer. Five random images of each of the cortex and the medulla per mouse were taken to analyze the pathological features in renal tissue. The Metamorph program was used to assess the area density for PSR, α-smooth muscle actin, CHOP, phosphorylated IRE1α, and F4/80 staining. This method involves setting a colour threshold in the Metamorph program to express the area density of the stain as a percentage of the image total area. The quantification of PAS staining was performed in the same program by manually selecting the casts and then calculating the percentage area of casts per image and then per mouse cortex or medulla. As well, glomerular scoring was assessed based on the scale and method used in previous studies^57^,^58^. CD3-stained sections were processed using the cell count tool in Image J software.

### RNA Isolation and NanoString Analysis

Total RNA was isolated from flash frozen mouse kidney tissue using the RNeasy Mini Kit (Qiagen). RNA integrity was assessed using the Agilent 2100 bioanalyzer and Agilent RNA 6000 Nano reagents (Agilent Technologies). Only RNA with RIN > 5 was used in our NanoString analyses. The code set for NanoString analysis was established based on literature searches of pathways that are important in determining therapeutic targets for the treatment of CKD. NanoString data was normalized against 7 housekeeping genes, IPO8, GUSB, TBP, YWHAZ, ACTB, GAPDH and RPLP2. P-values were corrected for multiple comparisons using the Benjamin-Hochberg procedure in R programming language and heat maps were produced using Java Treeview software. Fold changes were log-transformed and hierarchical gene clustering was performed on heat maps using Euclidean distance and complete linkage. On the heat maps, each column represents a time point or the effect of 4-PBA or CHOP deficiency on the 21 day Ang II/DOCA salt model. Each box represents the fold change for a particular gene in Ang II/DOCA salt-treated mice compared to their respective sham controls at the designated time point, treatment, or genotype manipulation. Fold changes were log_2_-transformed. Down-regulation is denoted by blue and up-regulation is denoted by red.

### Statistical Analysis

Data are shown as the mean ± standard error of the mean. Student’s T-test was used to analyze difference between groups. Differences among multiple groups were analyzed with analysis of variance followed by post-hoc Bonferroni tests in Graphpad Prism 5.0. A P-value of ≤0.05 was considered significant.

## Additional Information

**How to cite this article**: Mohammed-Ali, Z. *et al*. Endoplasmic reticulum stress inhibition attenuates hypertensive chronic kidney disease through reduction in proteinuria. *Sci. Rep.* **7**, 41572; doi: 10.1038/srep41572 (2017).

**Publisher's note:** Springer Nature remains neutral with regard to jurisdictional claims in published maps and institutional affiliations.
